# Physical literacy and health of Chinese medical students: the chain mediating role of physical activity and subjective well-being

**DOI:** 10.3389/fpubh.2024.1348743

**Published:** 2024-07-11

**Authors:** Xiaoqi Yang, Miaomiao Wang, Jiangong Wang, Shujie Zhang, Xiaoxia Yang, Liangyuan Zhao

**Affiliations:** ^1^School of Public Health, Shanxi Medical University, Taiyuan, Shanxi, China; ^2^Department of Exercise Rehabilitation, Shanxi Medical University, Taiyuan, Shanxi, China; ^3^Department of Management, Shanxi Medical University, Taiyuan, Shanxi, China

**Keywords:** physical literacy, health-related quality of life, physical activity, subjective well-being, health, Chinese medical students

## Abstract

**Background/objective:**

While Physical Literacy has been highlighted as a determinant in health in recent study, there is a dearth of studies examining its effect on physical health, and there is a little in the way of empirical data linking Physical Literacy to health outcomes. Accordingly, further empirical research is needed to clarify the mechanisms by which Physical Literacy affects physical health. The purpose of this study was to verify the role of medical students’ Physical Literacy on Health-related quality of life as well as to explore the chain mediating role of Physical Activity and Subjective Well-being in it.

**Methods:**

This study utilized a cross-sectional study design. The Physical Literacy, Health-related Quality of Life, Physical Activity ratings, and Subjective Well-being of students at Shanxi Medical University were all measured using an online survey administered in September 2023. A total of 1968 valid questionnaires were returned. First, descriptive statistics and correlation analysis were performed using SPSS software. Second, PROCESS was used to test the mediating role. Finally, we used structural equation modeling (Amos) to test the model fit.

**Results:**

There is a significant correlation between all variables. After mediation effects analysis, we found that there were three indirect pathways of physical literacy on health-related quality of life: a single mediating effect of physical activity, a single mediating effect of subjective well-being, and a chained mediating effect of physical activity-subjective well-being.

**Conclusion:**

The mediating role of physical activity and subjective well-being on the relationship between physical literacy and health-related quality of life has been confirmed. Our research results support the integration of physical literacy into physical education teaching and the modification of curriculum content by physical education teachers as part of efforts to enhance students’ physical activity levels, subjective well-being levels, and overall health. This study provides a new perspective for intervention in improving the health of medical students.

## 1 Introduction

College students’ emotional and physical well-being have been a source of worry in recent years. Researchers Grasdalsmoen et al. (1) observed a dose–response association between inactivity and negative mental health outcomes such self-harm and suicide attempts. Research by Vilugrón Aravena (2), Memon (3), and Wang et al. (4) shows that many modern college students engage in risky practices such eating poorly, sleeping in, using drugs and alcohol, not getting enough exercise, and so on. Further endangering their bodily and mental well-being are these vices. The proliferation of medical education has led to a heightened academic burden on medical students, resulting in significant health issues stemming from mental strain, sedentary behavior, inadequate nutrition, disruptions to circadian rhythms, and other unfavorable lifestyle practices. Furthermore, it is worth noting that medical students encounter elevated employment expectations and a rigorous test framework, factors that contribute to heightened psychological stress (5–9). Tension can have positive effects on students’ drive to learn in small doses, but in larger doses it can have negative physiological and psychological effects. Therefore, it is essential to undertake research into the wellbeing of medical school students as a whole and provide relevant recommendations for improvement. The Health-Related Quality of Life (HRQoL) questionnaire is a useful tool for gaining insight into and evaluating one’s overall health because of the breadth of topics it covers. This study evaluates the health status of medical students using the concept of HRQoL.

The multifaceted nature of physical literacy (PL) has been extensively acknowledged. Presently, it is widely defined to comprise the significance assigned to physical exercise during one’s lifespan, the acquisition and proficiency of information in this domain, as well as the inclination and drive to actively engage in physical activities (PA) (10–12). The first physical literacy assessment scale (PPLI) for adults appeared in 2016 (13), and has subsequently been revised and altered according to area, language and age, with all adapted PPLIs exhibiting satisfactory internal consistency and model fit. In 2012, Luo et al. (14) developed a college student physical literacy questionnaire (CSPLQ) for the college student population. Boldovskaia et al. (11) not only analyzed the internal consistency of this questionnaire, but also tested the simultaneous validity of the athleticism, physical condition, physical attractiveness, physical fitness, frequency of PA, and PA time variables. PL encompasses various components, including sports knowledge, sports awareness, physical fitness level, sports skills, and sports quality, etc., which can be produced under the influence of the acquired environment and physical education. A increasing body of research indicates that good PL is a precondition for active involvement in PA (15, 16), and there is already evidence that PL has a favorable impact on mental health (17). As a fundamental part of healthy living, PL is crucial to the upkeep and improvement of one’s health. The positive effects of PL on health are evident in many areas of daily life, including increased fitness and immunity, reduced risk of disease, better mental health, and reduced stress and anxiety (17–20). Therefore, the development of PL is crucial in the context of health improvement. While PL has been highlighted as a determinant in health in recent study, there is a dearth of studies examining its effect on physical health, and there is little in the way of empirical data linking PL to health outcomes. Accordingly, further empirical research is needed to clarify the mechanisms by which PL affects physical health.

PA refers to any bodily movement that is generated by skeletal muscles and necessitates the utilization of energy (21). More health benefits can be attributed to PA, such as improved muscular and bone strength, a more robust immune system, and better mental health (22–24). The World Health Organization (WHO) recommends that adults aged 18–64 should do at least 150–300 min of moderate-intensity aerobic PA throughout the week; or at least 75–150 min of vigorous-intensity aerobic PA throughout the week; or an equivalent combination of moderate- and vigorous-intensity activity (21). Students’ health and fitness can greatly benefit from regular PA. A person’s physical fitness, mental health, life satisfaction, immunity, developed willpower, and social adaptability can all benefit from engaging in sports (25). Previous research has indicated that PA can be classified into different levels of intensity, namely vigorous, moderate, and light intensity PA. Vigorous and light intensity PA can improve physical and mental health to some extent, but moderate intensity is most appropriate (26, 27). Light-intensity PA primarily contributes to the improvement of cardiorespiratory fitness. Moderate-intensity PA not only enhances cardiorespiratory fitness but also aids in weight reduction. In addition to improving cardiorespiratory fitness and reducing body weight, vigorous-intensity PA also promotes the development of muscle strength. By participating in sports activities, we can cultivate strong sports literacy, develop good exercise habits, and successfully improve our physical fitness and health. Therefore, boosting the participation of young individuals in PA will have major benefits for the future (28).

The adoption of Subjective Well-Being (SWB) as the dominant metric used to assess well-being has stimulated substantial scholarly investigation into human behavior, quality of life, and their antecedents. The term “SWB” is used to describe an individual’s level of contentment with their life as a whole, as well as their level of contentment in various aspects of their lives (29, 30). The enhancement of the body’s immune system can be facilitated by a state of well-being. Research has established a correlation between positive emotions, a sense of well-being, and heightened immunity, resulting in a decreased susceptibility to illness, expedited recovery from ailments, and reduced levels of stress. Furthermore, positive emotions have been found to alleviate stress and facilitate the healing process, thereby aiding individuals in recovering from significant stressors (31, 32). Engaging in sports activities has been demonstrated to help to the development of a powerful sense of accomplishment and self-efficacy. Because of this, an individual’s sense of self-worth and happiness improves (33, 34). Conversely, SWB is found to have a beneficial impact on an individual’s overall health. Nevertheless, the majority of research endeavors have primarily examined the correlation between SWB, sports literacy, and PA. Only a limited number of studies have specifically concentrated on the intermediary function of SWB within this particular framework.

Therefore, the aim of this study is to: (a) validate the role of medical students’ PL on quality of life. (b) explore the chain mediating role of PA and SWB in it. Our hypothesis posited that the PL of medical students would exert a favorable influence on their quality of life. Additionally, we proposed that PA and SWB would serve as crucial factors in mediating the relationship between PL and quality of life.

## 2 Materials and methods

### 2.1 Participants

The PL, quality of life, PA ratings, and SWB of students at Shanxi Medical University were all measured using an online survey administered in September 2023. Participants were selected using both a convenience sample and a snowball sample. All 2,200 surveys that were sent out were returned for analysis, for a perfect recovery rate. A total of 1968 questionnaires were considered genuine after excluding those with answer times over 600 s, below 120 s, or of low quality. This equated to a successful recovery rate of 89.5%. A total of 1968 people took part in the survey, with 828 (42.1%) men and 1,140 (57.0%) women. There were 1,264 (64.2% of the total) first-year students, 280 (14.2%) sophomores, 200 (10.2%) juniors, and 224 (11.4%) seniors or higher among the participants. Participants were from urban 744 (37.8%), town 596 (30.3%), and rural 628 (31.9%). The participants were all non-sports related majors. The age range of all subjects was 19.50 ± 7.30 years.

### 2.2 Procedure

The data were obtained using the web platform WeChat. Initially, the questionnaire was transformed into a web link to facilitate its distribution. The researchers then used WeChat’s private messages and WeChat groups to distribute the survey link, solicit participants, and collect data from the students in their WeChat address books. Participants were also asked to share the poll with their own WeChat contacts in order to maintain a steady stream of responses. The Ethics Committee at Shanxi Medical University gave their stamp of approval to this investigation.

### 2.3 Measures

#### 2.3.1 Measurement of physical literacy

Simplified Chinese version of the Perceived Physical Literacy Instrument (PPLI) is a reliable and valid instrument for measuring PL among Chinese college students. The 18-item scale, originally in Cantonese, was revised by Ruisi Ma et al. (35) to create an 8-item scale specifically designed to measure PL among college students in mainland China. Six hundred twenty-two college freshmen were split evenly into two groups for the study. Both exploratory and confirmatory factor analyses were conducted on subsets of the data. According to Whitehead’s definition (36), the scale encompasses three fundamental attributes: motivation, confidence and physical ability, and interaction with the environment. All three dimensions are interrelated and dependent on one another. The first three questions address motivation, whereas questions 4, 7, and 8 probe perceptions of one’s own physical skill and competency. The interaction with environment is addressed in questions 5 and 6. We use a 5-point Likert scale, from 1 (Strongly Disagree) to 5 (Strongly Agree), for every question. The total score for PL is obtained by summing the scores, resulting in a range of 8 to 40. The Cronbach’s alpha coefficient for this scale in this study was 0.943, the KMO value was 0.893, and the Bartlett’s spherical test value was 3,749. 707 (*p* < 0.001).

#### 2.3.2 Measurement of health-related quality of life

HRQoL was measured using the SF-12 Health Questionnaire Short Form (37). Somatic functioning (2 entries), somatic functioning’s influence on role functioning (2 entries), pain (1 entry), general self-assessment of health (1 entry), vitality (1 entry), social functioning (1 entry), the impact of emotions on role functioning (2 entries), and psychological well-being (2 entries) are the eight domains that make up this questionnaire. In particular, the first four domains deal with a person’s physical health, while the latter four deal with their mental health (38). Among them. The scoring system for somatic functioning ranged from 1 to 3, with descriptors ranging from “very limited” to “no limitation at all.” The remaining domains were scored on a scale of 1 to 5, with descriptors ranging from “no effect at all” to “very much,” “excellent” to “poor,” “all the time” to “never,” and so on. Reverse scoring applies to the entries with the numbers 1, 8, 9 and 11. The scores can be anywhere from 0 and 100, with higher numbers indicating a greater HRQoL. The Cronbach’s alpha number for this scale in this study was 0.846, the KMO value was 0.820, and the Bartlett’s spherical test value was 3665.507 (*p* < 0.001).

#### 2.3.3 Measurement of physical activity

The Physical Activity Rating Scale (PARS), as amended by Deqing Liang in 1994 (39), was utilized to evaluate the PA levels of the participants. The measure incorporates three factors: exercise intensity, time, and frequency. Multiplying the values of intensity, time, and frequency yields the formula for determining exercise score. Each factor is assigned a grade system consisting of five levels. Both intensity and frequency are given numerical values between 1 and 5 on a scale from 1 to 5. Point values for time range from 0 to 4, on a scale from 1 to 5. The final score for the workout might be anywhere from 0 to 100. The Cronbach’s alpha number for this scale in this study was 0.724, the KMO value was 0.661, and the Bartlett’s spherical test value was 309.704 (*p* < 0.001).

#### 2.3.4 Measurement of subjective well-being

This experiment exploited positive emotions and life satisfaction as evaluative indicators of SWB in order to undertake a comprehensive assessment. The Positive and Negative Affect Schedule (PANAS) (40) created by Watson et al. was used to measure positive affect. In addition, we assessed contentment with life using a modified version of the five-item Satisfaction with Life Scale (SWLS) developed by Chengqing Xiong et al. (41). The extent of affective experience was evaluated with a 5-point Likert scale that ranged from “very little” to “a great deal” for the Positive Affect Scale. A 7-point Likert scale was also used to rate participants on the Life Satisfaction Scale, with responses ranging from “strongly disagree” to “strongly agree.” In this study, the Cronbach’s alpha number for the Positive Affect Scale was 0.963, the KMO value was 0.946, and the Bartlett’s Spherical Test value was 5,952.688 (*p* < 0.001). The Life Satisfaction Scale had a Cronbach’s alpha of 0.930, a KMO value of 0.862, and a Bartlett’s Spherical Test value of 2599.545 (*p* < 0.001).

#### 2.3.5 Analyses

SPSS (27.0) and Amos (28.0) were used to organize and analyze the data statistically. At first, SPSS was used to calculate variable descriptive statistics. The interdependencies among the variables were also calculated. Next, we tested the mediating effect of PA and SWB using PROCESS (Model 6 justified by 5,000 bootstraps). In this study, we employed structural equation modeling (Amos) to assess the adequacy of the chain mediating effects of PA and SWB in the relationship between PL and HRQoL. The conditions for exponential fitting for structural equation modeling were χ^2^/df < 5, NFI > 0.8, IFI > 0.8, TLI > 0.8, CFI > 0.8, RMSEA <0.08.

## 3 Results

### 3.1 Common method bias test

A Harman one-way test was employed in order to mitigate the potential influence of common method bias and to ensure control over survey quality. The exploratory factor analysis yielded a set of seven components, each of which had eigenvalues greater than one. The percentage of explained variance for the first factor was 24.25%, which falls far below the essential threshold of 40% suggested by Podsakoff et al. (42). This finding indicates the absence of significant shared methodological bias within the scope of this investigation.

### 3.2 Descriptive statistics of variables

The averages and standard deviations of the participants’ PL, HRQoL, PA, and SWB were computed. Furthermore, Pearson correlation analyses were performed to examine the relationships between these variables. The outcomes of these analyses are presented in Table 1. The levels of HRQoL, PA, and SWB were all found to be significantly correlated with higher levels of PL. HRQoL was positively correlated with both PA and SWB in a substantial way. PA was found to be significantly correlated with SWB.

**Table 1:** Descriptive statistics of the applied measurement tools and their associations.

	Mean	SD	Physical literacy	HRQoL	Physical activity	Subjective Well-being
Physical Literacy	31.97	6.26	1			
HRQoL	45.92	6.52	0.593^**^	1		
Physical Activity	64.13	13.38	0.644^**^	0.694^**^	1	
Subjective Well-being	28.86	23.55	0.430^**^	0.397^**^	0.403^**^	1

**Correlation is significant at the 0.01 level (two tailed).

### 3.3 Analysis of the mediating role of physical activity and subjective well-being

**Figure 1:**
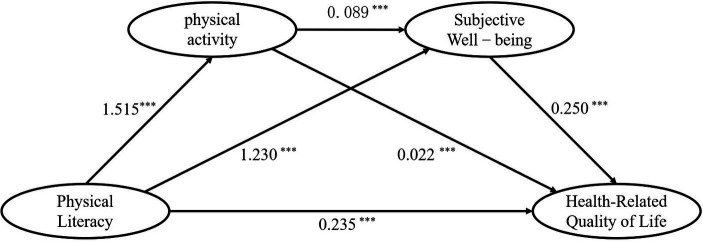
The chain mediating effect of physical activity and Subjective Well-being. ***p ≤ 0.001.

The PROCESS program suite utilized the MODEL6 chained mediator model. The reliability of the findings was evaluated by doing a bootstrap resample 5,000 times. The relationship between the independent variable of PL and the dependent variable of HRQoL was analyzed after controlling for three covariates: gender, grade level, and residential location. Additionally, PA and SWB were studied as mediator variables to determine their potential chained mediating effect in the relationship between PL and HRQoL. The results (see Table 2; Figure 1) indicate that PL exhibited a statistically significant predictive relationship with PA (*B* = 1.515, *t* = 10.592, *p* < 0.001); PL also exhibited a statistically significant predictive relationship with SWB (*B* = 1.230, *t* = 15.208, *p* < 0.001). When both PL and PA were used as predictors of SWB, the predictions yielded statistically significant results: PL (*B* = 1.230, *t* = 15.208, *p* < 0.001); and PA (*B* = 1.230, *t* = 15.208, *p* < 0.001). The variables PL (*B* = 0.235, *t* = 5.335, *p* < 0.001), PA (*B* = 0.022, *t* = 2.125, *p* < 0.001), and SWB (*B* = 0.250, *t* = 12.293, *p* < 0.001) collectively shown considerable predictive power in relation to Health-Related Quality of Life (HRQoL).

**Table 2:** The effect of physical literacy on HRQoL: Chain mediation regression analysis.

Outcome variable	Predictor variable	R^2^	F	B	SEs	t	LLCI	ULCI
Physical Activity	Physical Literacy	0.302	52.563	1.515	0.143	10.592	1.234	1.796
Subjective Well-being	Physical Literacy	0.439	76.142	1.230	0.081	15.208	1.071	1.389
	Physical Activity			0.089	0.023	3.831	0.043	0.134
HRQoL	Physical Literacy	0.527	89.925	0.235	0.044	5.335	0.148	0.322
	Physical Activity			0.022	0.011	2.125	0.002	0.043
	Subjective Well-being			0.250	0.020	12.293	0.210	0.290
HRQoL	Physical Literacy	0.361	68.823	0.610	0.038	16.092	0.535	0.684

### 3.4 Mediating effects test

Table 3 displays the results of the chain mediation analysis. The variable PL was revealed to have a substantial impact on the HRQoL with a coefficient of 0.235 (*t* = 5.335, *p* < 0.001, LLCI = 0.148, ULCI = 0.322). This proportion of the total effect was determined to be 38.5%. The combined impact of PA and SWB on PL and HRQoL was determined to have an indirect effect size of 0.375, accounting for approximately 61.5% of the overall effect size. Using Amos 28.0, we built a chain mediation model for structural equations. GFI = 0.927, NFI = 0.928, IFI = 0.949, TLI = 0.933, CFI = 0.948, and RMSEA = 0.068 were all within acceptable ranges for this model. The evidence presented indicates that the chain mediation model exhibits a strong fit index. Therefore, PL has a direct impact on HRQoL and an indirect impact on HRQoL through the promotion of increased PA and SWB. This study identified three indirect pathways through which PL affects HRQoL: a direct influence mediated by PA, a direct effect mediated by SWB, and a sequential effect mediated by the combination of PA and SWB. If you want the full scoop, look at Table 3.

**Table 3:** Test of chain mediation effect of physical literacy.

Influence path	Effect value	Boot SE	Boot LLCI	Boot ULCI	Relative mediation effect
Total effect	0.610	0.038	0.535	0.684	100%
Direct effect	0.235	0.044	0.148	0.322	38.5%
Total Indirect effect	0.375	0.047	0.286	0.468	61.5%
Physical Literacy →physical activity → HRQoL	0.034	0.017	0.002	0.067	9%
Physical Literacy →Subjective Well-being → HRQoL	0.307	0.046	0.223	0.402	82%
Physical Literacy →Physical Activity →Subjective Well-being →HRQoL	0.034	0.012	0.010	0.055	9%

## 4 Discussion

This study’s results suggest a link between young medical students’ reports of feeling lonely and their actual levels of physical and mental health. Initially, a favorable connection was identified between PL and HRQoL, which was somewhat influenced by PA and SWB. This study revealed a strong link between PA and HRQoL, underscoring the value of PL in supporting the physical well-being of young medical students. The importance of PL in promoting the mental health of young medical students is further highlighted by the positive correlation between SWB and HRQoL.

The present study has successfully identified that PA has a mediating role in the relationship between PL and HRQoL. Young medical students were shown to have a strong link between higher levels of PL and active participation in PA. PL and PA were shown to be positively related across the entire sample, with the β value of 1.515. This finding is consistent with another cross-sectional research. Yan et al. conducted a study which shown a strong positive relationship between self-reported perceived locus of control PL and both light PA (*β* = 0.097, *p* < 0.001) and vigorous PA (*β* = 0.172, *p* < 0.001) among a sample of 2,996 Chinese teenagers (43). In a sample of 1945 Chinese adolescents, Choi et al. found a positive link between self-assessed PL and self-reported length of PA, with the β value of 0.23 (44, 45). The researchers Melby et al. (46) and Coyne et al. (33) respectively discovered a correlation between PL and PA in a sample of 647 Danish children and 1,000 Canadian children, and the link was assessed by accelerometers or pedometers, with the β values 0.39 (33) and 0.18 (47). Paulina et al. (48) surveyed 1,518 Danish adolescents and found a significant positive relationship between PL and PA participation. In order to motivate people to take the necessary steps to improve their health, the Health Belief Model (48) stresses the importance of people being aware of the negative nature and severity of the consequences associated with unhealthy behaviors, as well as the significant advantages associated with engaging in positive health behaviors. PL serves as the fundamental basis for engaging in PA, exerting a favorable influence on motivation, self-assurance, and physical capacity to partake in such activities. Additionally, it fosters a more profound comprehension of the physiological and psychological advantages associated with PA, thereby influencing the intensity, duration, and frequency of engagement in such activities. A noteworthy positive association was observed between PA and HRQoL, as indicated by the β value of 0.022. However, it is important to note that the correlation coefficient was rather low, which could perhaps be attributed to the varying levels of activity associated with PA. The excessive burden placed on individuals in the context of PA can result in both physical and mental fatigue, ultimately leading to a decline in the quality of life. Insufficient levels of physical exercise can have a negative impact on an individual’s quality of life, leading to suboptimal levels of HRQoL. In order to promote health, it is necessary to individualize the intensity, duration, and frequency of PA in accordance with the guidelines set forth by WHO. Additionally, the amount of exercise should be suitably modified based on an individual’s physical condition to attain the desired health benefits. Consequently, a high level of PL will better guide students to participate in PA and promote health.

First, this research shows that SWB mediates the relationship between PL and HRQoL. The data imply that PL has a positive effect on young people’s psychological well-being. Consistent with prior cross-sectional design research, we found a positive connection between PL and SWB (*β* = 1.230). Melby et al. (45) surveyed 1,518 Danish adolescents and found that there was a positive correlation between PL and each of the five factors that make up happiness. Ma et al. surveyed a group of 5,265 Chinese university students. Consistent with earlier studies (15, 33, 46), the results showed that PL emerged as a significant predictor of mental health. PL comprises various factors such as self-confidence, physical functioning, and connection with the environment. These factors contribute to the enhancement of an individual’s confidence, athleticism, and social skills, ultimately leading to an improvement in SWB. Additionally, SWB serves as a mediating variable between PL and HRQoL, with PL exerting a positive influence on HRQoL by means of SWB. Based on the emotional consistency hypothesis, individuals with positive emotions will be more inclined to perceive, notice, interpret, and judge emotional information pleasantly (49). The findings of this study indicate that there is a correlation between happy emotions and a greater HRQoL. Individuals with elevated levels of PL demonstrate enhanced self-assurance, an optimistic attitude, and effective communication abilities. They also have a lower risk of developing mental health problems thanks to their exceptional social skills.

Furthermore, this study additionally demonstrated a mediation mechanism between PA and SWB in PL and HRQoL. On one side, PA significantly and positively predicted SWB, aligning with findings from previous cross-sectional research studies. Both studies conducted by Wang et al. and Marques et al. showed a positive correlation between PA and SWB. Firstly, PA can alleviate individuals’ stress and promote mental health. Engaging in PA has the potential to temporarily alleviate the stress associated with work and study, divert individuals’ attention toward sports, and facilitate both physical and mental relaxation (50). Additionally, PA can facilitate social engagement and foster the development of positive interpersonal connections. Engaging in social interactions can enhance an individual’s sense of social support and belonging, hence positively influencing their SWB (51–54). Meanwhile, PA can also help people develop good habits. Engagement in PA enables individuals to develop a comprehensive comprehension of their physical well-being, hence augmenting their health consciousness. Subsequently, individuals should prioritize the cultivation of a wholesome lifestyle, which has a great effect on the mental health of individuals and improves their SWB (46, 55). Nevertheless, the beta coefficient for PA in predicting SWB was found to be just 0.089, indicating a weak positive relationship. This modest connection may perhaps be attributed to the extent of engagement in PA. In the long run, physical health problems caused by physical inactivity might have a detrimental impact on students’ future levels of productivity and overall state of well-being (56). Insufficient PA can also have a substantial impact on students’ scholastic achievement and productivity (57). However, considering the prevailing global insufficiency of PA, we doubt whether high-intensity PA still has a positive impact on students’ s SWB and health-related quality of life. Engaging in high-intensity sports activities unavoidably leads to bodily overload, which, over time, can result in physical weariness. This fatigue can subsequently impact emotional well-being and potentially have adverse effects on both physical and mental health. On the other hand, HRQoL is also a process of interaction that involves the dynamic interplay between an individual’s motor capacity, motivation, and the surrounding environment. Hence, the results of our study indicate that PL has an impact on HRQoL through PA and SWB. Additionally, PL can influence HRQoL by mediating the relationship between PA and SWB. These findings provide further insights into the underlying mechanisms that affect the health of medical students.

### 4.1 Implications

Enhancing the PL of medical students is crucial. It is imperative to conduct an assessment of students’ PL upon commencement of their initial year at the institution. Also, lectures on PL and PA should be provided in order to enhance their comprehension of these subjects. In the future, students can arrange their time for educational pursuits and engage in a state of well-being conducive to enhancing their learning efficacy. Additionally, it is vital to provide information to parents at school. The lifestyles of individuals are significantly shaped by their surroundings, and the prevailing sporting culture within a family group plays a crucial role in determining a student’s engagement in PA. Establishing an optimal sports milieu for students both the family and school settings can significantly facilitate the cultivation of students’ authentic comprehension of sport, engagement in sporting activities, and enduring consciousness of the significance of sport throughout their lives.

### 4.2 Limitations

This study does have some limitations. First, primary data in this research was obtained by means of a questionnaire. During the process of responding to the questionnaire, it is possible that certain students may exhibit bias or tend to overestimate their PA situation when recalling their experiences. In following research endeavors, it is possible to employ measurement equipment such as accelerometers to effectively record PA data. Furthermore, the individuals involved in this research were Chinese university students. There is a worldwide problem with college students not engaging in enough PA, and national differences in education systems contribute to varying cultural attitudes about physical education. As a result, these differences could play a role in the varying degrees to which various groups are impacted by and react to this issue. Further research can be undertaken involving diverse populations.

## 5 Conclusion

This study is important because there is a lack of cross-sectional studies that investigate the link between self-reported PL and health. The findings of this study indicate that PA and SWB serve as partial mediators in the association between PL and HRQoL. The variables of SWB and LPA have been identified as potential factors that may contribute to the impact of positive psychology interventions on HRQoL. Additionally, it has been observed that PA has a mediating function in the relationship between SWB and both positive psychology interventions and HRQoL. Based on the findings of the study, the integration of PL into physical education, along with modifications made by physical education teachers to the curriculum content, has been shown to improve students’ PA levels and SWB. This improvement in PA and SWB has the potential to positively impact students’ physical and mental health, as well as their academic performance, overall quality of life, and future professional endeavors.

## Data availability statement

The raw data supporting the conclusions of this article will be made available by the authors, without undue reservation.

## Ethics statement

The studies involving humans were approved by Ethics Committee of Shanxi Medical University. The studies were conducted in accordance with the local legislation and institutional requirements. The participants provided their written informed consent to participate in this study.

## Author contributions

XqY: Writing – original draft. MmW: Writing – original draft. JgW: Writing – original draft. SjZ: Writing – original draft. XxY: Writing – review & editing. LyZ: Writing – review & editing.
